# Latex Allergy in Children

**DOI:** 10.3390/jcm13010124

**Published:** 2023-12-25

**Authors:** Stefania Arasi, Simona Barni, Lucia Caminiti, Riccardo Castagnoli, Mattia Giovannini, Lucia Liotti, Carla Mastrorilli, Francesca Mori, Luca Pecoraro, Francesca Saretta, Mariannita Gelsomino, Angela Klain, Michele Miraglia del Giudice, Elio Novembre

**Affiliations:** 1 Pediatric Allergology Unit, Allergy Diseases Research Area, Bambino Gesù Children’s Hospital IRCCS, 00165 Rome, Italy; stefania.arasi@opbg.net; 2 Allergy Unit, Meyer Children’s Hospital IRCCS, 50139 Florence, Italy; simonabarni@hotmail.com (S.B.); mattiag88@hotmail.it (M.G.); francesca.mori@unifi.it (F.M.); elio.novembre@unifi.it (E.N.); 3 Allergy Unit, Department of Pediatrics, AOU Policlinico Gaetano Martino, 98124 Messina, Italy; lucycaminiti@yahoo.it; 4 Department of Clinical, Surgical, Diagnostic and Pediatric Sciences, University of Pavia, 27100 Pavia, Italy; riccardo.castagnoli@yahoo.it; 5 Department of Health Sciences, University of Florence, 50139 Florence, Italy; 6 Pediatric Unit, Department of Mother and Child Health, Salesi Children’s Hospital, 60123 Ancona, Italy; lucialiotti@libero.it; 7 Pediatric and Emergency Department, Pediatric Hospital Giovanni XXIII, AOU Policlinic of Bari, 70126 Bari, Italy; carla.mastrorilli@gmail.com; 8 Pediatric Unit, Department of Surgical Sciences, Dentistry, Gynecology and Pediatrics, University of Verona, 37126 Verona, Italy; luca.pecoraro@aovr.veneto.it; 9 Pediatric Department, Latisana-Palmanova Hospital, Azienda Sanitaria Universitaria Friuli Centrale, 33100 Udine, Italy; francescasaretta@gmail.com; 10 Pediatric Allergy Unit, Department of Life Sciences and Public Health, Fondazione Policlinico Universitario A. Gemelli IRCCS, Catholic University of the Sacred Heart, 00168 Rome, Italy; 11 Department of Woman, Child and General and Specialized Surgery, University of Campania Luigi Vanvitelli, 80138 Naples, Italy; klainangela95@gmail.com (A.K.); michele.miragliadelgiudice@unicampania.it (M.M.d.G.)

**Keywords:** latex allergy, molecular diagnostics, management, pediatrics, prevention, spina bifida

## Abstract

Notwithstanding the efforts made in the last decades to mitigate the consequences of natural rubber latex allergy, this disease continues to be a major health problem, especially in developing countries. The categories of patients with greater and frequent exposure to latex (such as health care professionals and, in the pediatric field, subjects who undergo repeated surgery, e.g., those suffering from spina bifida and urogenital malformations) have an increased risk of developing sensitization and allergy to latex. Herein we provide an overview of the current knowledge and practical recommendations with a focus on epidemiology, diagnostics, and management (including both prevention and therapy) in order to guide a correct recognition and containment of this potentially fatal condition.

## 1. Introduction

The term “latex” derives from “leche”, the Spanish word for milk, and describes the milky white sap secreted from an incised trunk. Thousands of plants produce this milky liquid, but nowadays the sap of the rubber tree, Hevea brasiliensis, native to the Amazon, is the main source of commercially produced natural rubber latex (NRL) [1]. Around 1840, the vulcanization process was developed, which solved the problem of rubber objects’ instability and allowed for the production of flexible materials, primarily used in the medical field, including gloves, catheters, tourniquets, condoms, etc. All these products contain numerous latex proteins capable of inducing an immune response in sensitized individuals. The first two cases of immediate latex reactions were reported in Germany in 1927, but the first true report dates back to 1979 when an urticarial immediate reaction after contact with latex gloves was described [1]. Subsequently, and until the 1990s, many other cases were described [2]. The increased exposure to latex products, especially gloves in healthcare settings and the food industry, has been the determining factor in the increased prevalence of the disease. Consequently, scientific interest, as evidenced by the number of international publications, has also grown in parallel, peaking in the late 1990s [1]. The production of low-allergenicity gloves, the reduction in use or banning of latex gloves in some countries, and targeted public health campaigns have led to a significant reduction in latex allergy in subsequent years. However, global natural rubber production remains high and is even increasing. In recent years, there has been widespread use of products containing latex in homes, schools, and workplaces (Table 1). In fact, in 2020, latex production amounted to approximately 13 million tonnes, compared to 6.8 tonnes in 2000. Therefore, the disease continues to be a global public health problem [3] and must be recognized and adequately treated.

The purpose of this bibliographic study is to provide an overview of the current knowledge and practical recommendations with a focus on epidemiology, diagnostics, and management (including both prevention and therapy) in order to guide a correct recognition and containment of this potentially fatal condition.

## 2. Epidemiology and Risk Factors

Latex allergy can affect both children and adults, although there is paucity of data on the pediatric population. The reported prevalence varies greatly depending upon the population studied and the methods used to detect sensitization. Karila et al. [4] reported in their survey (1989–2001) that latex was the second most common cause of preoperative accidents (27%) in pediatric populations. Exposure to latex-derived products during surgery, either through skin contact or by inhalation, has been described as risk factor for developing latex allergy. In fact, the prevalence rate of latex sensitization and allergy is closely associated with the degree of exposure, especially in individuals exposed for occupational reasons, such as healthcare workers, or for medical issues. The population at greatest risk of latex sensitization and allergy includes patients undergoing repeated surgical interventions (e.g., patients with spina bifida or other malformations) or repeated anesthesia and catheterizations (e.g., patients with urogenital anomalies, cloacal anomalies, and insulin-treated diabetics) [3,5,6,7,8,9]. Patients with history of more than five surgeries have a higher risk of presenting latex sensitization [9]. Many of these medical conditions have their onset at pediatric age (Table 2). In particular, the highest prevalence of latex sensitization has been reported in pediatric patients with spina bifida, ranging from 26% [10] to 47.9% [8,11,12,13]. By implementing latex-free measures from birth in children with spina bifida, compared to historical controls, it has been found that the prevalence of latex sensitization has decreased. Specifically, in a Spanish study by Nieto et al. [10] comparing 15 children with spina bifida before the introduction of preventive measures in 1994 and 22 children born after that date, the prevalence of latex sensitization decreased from 26.7% to 4.5% [10]. In a German study by Blumchen et al. [12] comparing 120 children with spina bifida who underwent surgery after the introduction of latex-free measures with 87 similarly aged children who were operated on earlier without prevention, the prevalence of latex sensitization decreased from 55% to 5% (Table 3).

Healthcare workers (such as doctors, nurses, dentists, biologists, ultrasound technicians, midwives) were the professional group most affected by latex allergy due to the frequent and continuous use of latex gloves in the 1980s and 1990s to prevent the transmission of infectious diseases such as the human immunodeficiency virus (HIV) and hepatitis C virus (HCV) [14,15,16] during the epidemic peak of those years. They were exposed to latex through contact with the allergenic content of gloves as well as through inhalation of airborne allergens released from the starch powder present in gloves.

Moreover, during the recent COVID-19 pandemic, the massive use of personal protective equipment provoked severe adverse reactions in latex allergy patients and negatively affected their quality of life [17].

Despite this, in recent decades, with the introduction of preventive measures, sensitization among healthcare workers has gradually decreased [18,19] (Table 4).

In particular, a study conducted in a tertiary dermatology center in Denmark showed that the prevalence of sensitization to natural rubber latex decreased from 6.1% in 2002–2005 to 1.9% in 2006–2009 and further to 1.2% in 2010–2013 (*p* < 0.0001). The prevalence of clinical latex allergy also decreased from 1.3% in 2002–2005 to 0.5–0.6% in 2006–2013 (*p* < 0.004) [19].

However, the risk of sensitization and allergy remains significant, especially in countries that lack adequate resources for implementing preventive measures or where there is a risk of further exposure to other latex-containing products [8]. According to a study conducted by Wu et al., the prevalence of latex allergy and sensitization among healthcare workers is 9.7% and 12.4%, respectively. This study analyzed data from studies conducted in different countries between 2009 and 2015, involving a total of 19,233 participants [4].

An aggregate analysis of 11 epidemiological surveys published between 2006 and 2015, including studies from various countries (also developing countries), showed a significantly lower but still significant prevalence of latex sensitization (5.1%) and latex allergy (4.2%) among healthcare personnel [20]. A recent Argentine study showed a latex sensitization prevalence of 7.96% (95% confidence interval: 3.70–14.58) among physicians in a children’s hospital [21]. Latex allergy is an emerging problem in developing countries as well: recent studies reported latex hypersensitivity in 9.1% of healthcare workers in South India [22] and in 9.2% of Turkish healthcare workers [23].

In addition to healthcare workers, other occupational groups, including rubber industry workers, hairdressers, homemakers, researchers working in biology or chemistry, gardeners, and food handlers, are at high risk of developing latex allergy [3,24,25]. 

Atopy has also been reported as a risk factor for developing latex sensitization and allergy. In the general population of children, the percentage of latex sensitization is approximately 1% [4], but it increases to 3–5% in atopic children [26,27,28], with about half of them experiencing clinical manifestations (Table 3).

Other factors that have been associated with a higher risk include genetic factors (HLA-DR phenotype and polymorphisms in the interleukin-13 and interleukin-18 promoters) [29] and hand dermatitis, which, due to barrier impairment, facilitates the passage of latex allergens [20].

It is worth noting that latex allergy is not exclusive to the aforementioned professional groups. There is evidence that the general population without occupational latex exposure can develop allergic sensitization and, therefore, latex allergy [30].

In conclusion, latex allergy remains an important condition from an epidemiological perspective due to the wide range of products that contain latex [8] and the challenges faced by many countries in adopting effective prevention measures.

**Table 2:** Clinical conditions associated with an increased risk of latex sensitization.

Diseases Associated with an Increased Risk of Sensitization to Latex	Sensitization to Latex	Author, Year	Country	Ref.
Spina bifida	26%	Nieto, 2002	Spain	[10]
	47.9%	Cremer, 2011	Germany	[11]
Urogenital anomalies	16.9%	Cremer, 2011	Germany	[11]
Anorectal malformations	-	-	-	-
Tracheoesophageal fistula	17%	Cremer, 2011	Germany	[11]
Multiple congenital anomalies	-	-	-	-
Ventriculoperitoneal shunt	-	-	-	-
Cerebral palsy	-	-	-	-
Quadriplegia	-	-	-	-
Prematurity	-	-	-	-
Atopy	3.8%	Jorge, 2006	Portugal	[27]
	4%	El-Sayed, 2014	Egypt	[28]

**Table 3:** Prevalence of latex sensitization and allergy in the general pediatric population and those exposed to risk factors.

Studied Population	Latex Allergy	Sensitization to Latex	Country	Author, Year	Ref.
General Population	4.3%	2.1%	Worldide	Wu, 2016	[8]
Patients with spina bifida	46%	-	Singapore	Chua, 2013	[13]
		47.9%	Germany	Cremer, 2011	[11]
	37%	55%	Germany	Blumchen, 2010	[12]
		26.7%	Spain	Nieto, 2002	[10]
Patients with spina bifida operated in a latex-free environment		4.5%	Spain	Nieto, 2002	[10]
	0.8%	5%	Germany	Blumchen, 2010	[12]
Patients with myelomeningocele	20%	25%	Brazil	Bueno de Sa, 2013	[5]
	19.5%	-	Spain	Parisi, 2016	[9]
Atopic Patients	2.6%	3.9%	Italy	Meglio, 2002	[26]
	0.5%	3.8%	Portugal	Jorge, 2006	[27]
	2.7%	4%	Egypt	El-Sayed, 2014	[28]

**Table 4:** Latex allergy in healthcare workers.

	Sensitization to Latex	Country	Author, Year	Ref.
Doctors and other healthcare workers	5.1%	Belgium	Vandenplas, 2017	[20]
	7.9%	Argentina	Laurino, 2020	[21]
	9.1%	India	Sakkaravarthi, 2022	[22]
	9.2%	Turkey	Aksoy, 2023	[23]

## 3. Allergens and Allergic Sensitization 

Natural latex is composed of water (55–65%), cis-1,4-polyisoprene rubber (34%), sugars (1.0–2.0%), sterol glycosides (0.1–0.5%), resins (1.5–3.5%), ash (0.5–1.0%), and, finally, proteins (2–3%) [31]. The allergenic components of latex (Hevea brasiliensis, Hev b 1–15), officially included in the International Nomenclature Committee of Allergens (IUIS) nomenclature are presented in Table 5 [32,33]. For a correct interpretation of the terminology, please refer to BOX 1. The principal allergenic components of latex are listed below:

- Hev b 1, or latex elongation factor, is a 14 kDa protein involved in the synthesis of polyisoprene. It is a major allergen in patients with spina bifida and a minor allergen in healthcare workers. Being insoluble in water, its availability for inhalation is low.
- Hev b 2 is a 34 kDa secondary allergen belonging to the group of plant defense proteins. Depending on the geographic region, 5% to 15% of allergic patients are sensitized to it. No differences in sensitization have been observed between patients undergoing surgery and healthcare workers.
- Hev b 3 belongs to the group of rubber particles and has a molecular weight of 24–27 kDa. It shares its biological function with Hev b 1, and, like Hev b 1, it is insoluble and represents the main allergen in patients with spina bifida.
- Hev b 5 is a 16 kDa acidic structural protein whose biological function is unknown. It is the main allergen in various high-risk groups, being found in 92% of healthcare workers and 56% of patients with spina bifida. Its prevalence varies from region to region for reasons that remain unclear. Hev b 5 shows multiple isoforms and exists in very small quantities in non-amino acid extracts, such as those used in diagnosis. Hev b 5 shows homology with the acidic protein in kiwi and other fruits.
- Hev b 6, or prohevein (precursor of hevein, Hev b 6.01), is a 20 kDa allergen belonging to class I chitinases. It has a defensive function as it degrades chitin, a component of fungal cell walls and insect exoskeletons. Processing leads to two allergenic fragments, the N-terminal, or hevein (Hev b 6.02), and the C-terminal (Hev b 6.03), which act independently. Hevein is the more important of the two and represents a major allergen in healthcare workers compared to patients with spina bifida. Its sequence shows >50% identity with fruit chitinases such as banana, avocado, and chestnut, giving rise to the so-called “latex-fruit syndrome”, which is included in the cross-reactivity syndromes between latex and fruit allergens [29].
- Hev b 7 is a 43 kDa protein that is more than 50% homologous to patatin, a storage protein in Solanaceae, thus explaining cross-reactivity with these plants. Hev b 7 is recognized by 23% to 45% of patients and is therefore a relevant allergen but not a major one.

Traditionally, tests for the specific IgE (sIgE) evaluation of latex were based on quantifying serum sIgE directed against crude natural allergen extracts. However, a positive sIgE for crude extracts should always be interpreted with caution, as it may simply reflect cross-sensitization rather than true allergy. For example, in the case of latex, it has been shown that ubiquitous structures such as α-1,3-fucose and β-1,2-xylose (which are characterized by cross-reactive carbohydrate determinants (CCDs) present on plant glycoproteins), α-1,3-fucose-containing CCDs from hymenoptera venom glycoproteins, and plant profilins can lead to false positive results [34,35]. 

Therefore, sIgE testing should not be used alone to diagnose IgE-mediated latex allergy but should be accompanied by the so-called component-resolved diagnosis (CRD), which relies on sIgE antibodies directed against individual components purified from natural sources or produced with recombinant techniques, rather than crude extracts obtained from native allergens [36]. CRD enables better discrimination between clinically irrelevant sensitization and allergy and enables the creation of personalized sensitization profiles [37].

The commercially available specific IgE tests for latex components are generally non-glycosylated recombinant proteins (r) Hev b 1, 3, 5, 6.01, 6.02, 8, 9, and 11 [38]. 

Particularly, rHev b 5 and 6, and to a lesser extent rHev b 1 and 3 (both proteins associated with rubber particles), are the most important biomarkers for diagnosing IgE-mediated latex allergy. Sensitization to Hev b 5 and 6 is mainly found in healthcare workers and to a lesser extent in children with spina bifida and meningomyelocele. Conversely, some patients, especially children with spina bifida, are sensitized to Hev b 1 and Hev b 3, which are almost exclusively present in rubber-producing plants and therefore less commonly responsible for cross-reactivity with homologous allergens present in fruits/pollens (and responsible for the so-called latex-fruit syndrome) [39]. On the other hand, sensitization to Hev b 8 (profilin) generally, although not always, indicates clinically irrelevant cross-reactivity [36]. 

Supporting the aforementioned observations, in a large case series reported by Ebo et al. [37], the diagnosis of IgE-mediated latex allergy in all patients was defined by the combination of Hev b 1, 3, 5, and 6.02. Over three-quarters of the patients tested positive for Hev b 5 and/or 6.02. A limited number also showed positivity for Hev b 1 and/or Hev b 3. In contrast, none of the individuals showing clinically irrelevant latex sensitization tested positive for any of these components, but 75% of them presented positivity for Hev b 8. Additionally, it is important to note that since all available latex components are non-glycosylated proteins, they constitute a useful tool for discriminating clinically irrelevant positive sIgE results for latex resulting from sensitization to the CCDs of plant and invertebrate origin.

Therefore, patients suffering from IgE-mediated latex allergy may exhibit distinct sensitization profiles and clinical phenotypes, which will be described in more detail below.

Routes of exposure to latex allergens: Since latex allergy affects not only healthcare professionals and those who frequently use latex gloves but also the general population without occupational exposure to latex products, it is important to be aware of the possible routes of exposure to latex allergens [8].

- Direct skin contact: Direct contact of the skin with latex-derived products is the main route for the development of latex allergy. Studies on healthcare workers have suggested that latex sensitivity appears to increase with the duration of exposure [8]. In addition to gloves and medical devices containing latex, which have received significant attention, thousands of products may contain natural rubber latex, which can be present in the product itself or its packaging or introduced during the manufacturing process or storage.
- Direct contact with mucous membranes: Patients with spina bifida or those undergoing multiple surgeries will be sensitized by the direct contact of latex-containing devices with their body fluids and mucous membranes [8,40].

As previously specified, while Hev b 5 and Hev b 6 (particularly the hevein domain Hev b 6.02) have been recognized as major allergens in healthcare workers, patients with spina bifida primarily exhibit IgE reactivity to Hev b 1, Hev b 3, and Hev b 5.

- Contact can also be indirect, occurring, for example, through the ingestion of food that has been handled by a worker wearing latex gloves or by having contact with a person who has been blowing up balloons.
- Airborne exposure to latex: Latex allergens present in the air can be inhaled and cause allergic reactions. Two of the main sources of inhalable latex allergens include cornstarch particles used in powdered latex gloves and tire dust (especially for those living near busy roads) [8]. To prevent sticking, latex gloves were typically produced by adding cornstarch particles as powder. Latex allergenic proteins can attach to the dust particles and disperse into the air, causing allergic reactions. It has been shown that the introduction of powder-free gloves significantly reduces the prevalence of latex allergy (see “Management: prevention and therapy”).
- Latex contamination in food and medications.
  It has been reported that latex allergies can also be caused by:
  - Food contaminated by workers wearing latex gloves.
  - Medications/vaccines contaminated by containers or medical devices containing latex. Natural rubber is widely used in food additives, packaging, and medical devices [35].

The Centers for Disease Control and Prevention (CDC) has compiled an updated list of vaccine packaging that contains latex [41].

- Cross-reactivity with plant-based foods.

Tropical fruits (such as avocado, papaya, passion fruit, mango, and pineapple), as well as fruits such as banana, fig, melon, peach, chestnut, and kiwi, contain proteins that can cause cross-reactions with latex [38]. Additionally, some vegetables such as tomatoes and potatoes can induce cross-reactivity. [42].

In conclusion, the severity of latex allergy significantly depends on the specific molecular allergenic component involved, as well as the route, degree, and number of exposures. Direct and massive contact of latex with mucous membranes is associated with the highest percentage of individuals who become sensitized and exhibit even severe symptoms of latex allergy (including anaphylaxis) [43].

## 4. Clinical Manifestations and Cross-Reactions

Clinical manifestations associated with latex exposure are various, and both immunological and non-immunological mechanisms can be identified underlying them [44]. Non-immunological mechanisms can cause localized dermatitis with itching, dryness, and redness in areas of contact with latex, where latex acts as an irritant. Reactions based on immunological mechanisms can be both type I (IgE-mediated) and type IV (cell-mediated) (Table 6).

The allergen can cause a reaction through various routes. Parenteral exposure is associated with more severe and sudden reactions such as anaphylactic shock, which can appear a few minutes after exposure to latex [45]. When contact with the allergen occurs by inhalation, rhinoconjunctivitis, widespread urticaria, bronchospasm, and, in some cases, anaphylaxis may occur. Among patients of pediatric age, Kimata reported nine cases of latex allergy in infants less than 1 year of age who experienced facial edema or rash, bronchospasm, or anaphylaxis after exposure to latex. All children had a positive SPT for latex; the contact with the allergen occurred through the oral mucosa (pacifier, teat, balloon), nasal (nasal lavage device), or anal (enema cannula) [46]. Anaphylaxis is the most serious clinical manifestation and can be potentially fatal, leading to death from respiratory failure and/or cardiocirculatory shock. In cases of intraoperative anaphylaxis, latex allergy should always be investigated. The diagnosis of these forms is often complex, and although in recent years, the percentage of intraoperative anaphylaxis attributable to latex allergy has been decreasing [47], it must be placed in a differential diagnosis with other potential intraoperative triggers such as anesthetic drugs, muscle relaxants, and antibiotics. In a recent review, Ma et al. analyzed 21 cases of intraoperative anaphylaxis in children; in seven cases, the allergen involved was latex [48]. Delayed immune-mediated reactions (type IV) can be associated with latex additives or with the latex itself. These contact reactions often involve the hands and are due to the use of latex gloves. Perioral dermatitis may also occur in children undergoing repeated dental surgery. Latex is, in fact, reported among the main allergens in contact dermatitis in children. Clinical pictures of this type can also occur after the use of various objects such as shin guards, diapers, or clothing [49].

In these delayed forms, clinical pictures are highlighted, which may include itching, hyperemia, vesicles, crusted lesions, desquamation followed by a state of persistent skin dryness, and lichenification.

A phenomenon of cross-reactivity between the latex and various allergens of the plant world linked to the recognition of structurally similar epitopes (cross-sensitization) can be detected. The main cross-reactive allergens involved have been identified in profilins (Hev b 8), patatins (Hev b 7 patatin-like and Sol t 1 of potato), PR-3 (Hev b 6.02 and class I chitinase), PR-2 (Hev b 2 β-1,3-glucanase of latex and pepper L-ascorbate peroxidase), and PR-14 (lipid transfer proteins). Latex heveins, especially Hev b 6.02, have approximately 70% homology with class I chitinases [50]. It is, therefore, possible that subjects sensitized to latex simultaneously have sensitizations to the chitinases present in some foods, as they are thermolabile and rapidly degraded at the gastric level. This phenomenon explains the reactions mainly localized in the oral area and limited to uncooked foods such as fruit and vegetables. The association between these detected cross-sensitizations and their clinical impact is not always clear. A total of 30–50% of patients with latex allergy show hypersensitivity to foods of plant origin, especially if eaten fresh. In 1994, Blanco et al., [51] observed that in a group of 25 patients with latex allergy, about 50% had one or more hypersensitivity to vegetable foods, which they defined with the term latex-fruit syndrome (LFS). Numerous studies have reported cross-reactivities with latex allergens [3,52,53,54]. The latex allergens reported to date in cross-reactivity cases are shown in Table 7.

A large number of fruits and vegetables have been associated with LFS. This connection is based on clinical evaluations, positive skin tests, and the identification of cross-reactive components through the use of natural or recombinant allergens (e.g., chestnut, kiwi, avocado, banana, tomato, passion fruit, papaya, mango, celery, pepper, etc.). Ricci et al. [55] studied an Italian pediatric population of 22 latex-allergic patients, 36% of whom had LFS. Kiwi was the most reported food, followed by chestnut, peach, melon, cherry, and apple. The authors found a statistically significant prevalence of LFS in patients with moderate-to-severe latex allergy compared to those with mild clinical pictures. In a recent multicenter study by Takemura et al. [56], 97 children with a history of fresh fruit allergy were enrolled. Of these, 76% had an allergy associated with oral allergy syndrome. The results of the work show a significant association between the specific IgE of latex and Bet v 2 profilin. In the population studied, only 3% of the children had a previous history of latex allergy, and the increase in latex IgE was associated with the family of Hev b 8 profilins that act through a cross-reactivity mechanism. This allergological aspect is constantly evolving, as demonstrated by the recent discovery of a newly described protein of peach and apricot called ENEA (Pru ar 5) [57], which presents a cross-reactivity with the Hev b 5 protein of the latex and with protein Man 5 and [58] cassava. Its concentration inside the peach is variable, and this could explain the variability in the allergic reactions that can occur in this context. Hev b 5 latex protein can also cross-react with spices such as curry [59]. To date, the list of plant foods involved in cross-reactions with latex has greatly increased compared to the first reports, which only involved tropical fruits, and includes, in addition to fresh fruit, vegetables, dried fruit, and cereals such as cassava. For this reason, the literature increasingly speaks of latex-associated food allergy in the description of this clinical picture.

Cross-sensitization can also occur with some pollens, such as those of grasses, and with some ornamental plants, such as *Ficus benjamin* and *Euphorbia pulcherrima* (Christmas star) [60]. In a pediatric study by Casquete-Roman et al. [61], sera from 106 children with pollen allergies and clinical histories of associated respiratory manifestations were analyzed. No patient enrolled in the study presented signs and symptoms upon exposure to latex. Approximately half of the children in the population studied had sensitization to latex. The authors report that children sensitized to grass profilins (Phl p 12) were positive for latex profilins (Hev b 8). Thus, latex sensitization in this group of patients is attributable to the cross-recognition of grass pollen profiling. A few patients have recently been identified with an IgE-mediated latex allergy apparently associated with Hev b 12 (non-specific latex lipid transport protein) monosensitization. The main reasons for monosensitization to Hev b 12 remain undefined but could, to some extent, relate to an underlying allergy to *Cannabis sativa* [62,63].

Therefore, molecular diagnostics help us to identify the relevant allergens in cases of allergic reaction and cross-sensitizations that have no real clinical confirmation. Orienting within the cross-sensitization of latex proteins is not easy considering that we do not know all the proteins involved, and we could, therefore, run into diagnostic errors, prescribe unnecessary elimination diets, or erroneously consider a patient as allergic to latex. It is therefore necessary to define each patient’s sensitization profile, which, together with the clinical history, guides us in defining the correct allergy management. Diagnostic algorithms have been proposed in the literature for better management of cross-reactivity in latex allergy [33].

**Table 7:** Latex allergens with reported cases of cross-reactivity.

Allergen	Cross-Sensitization	Author, Year Ref.
Hev b 1 (rubber elongation factor)	Papain	Baur, 1995 [52]
Hev b 2 (beta 1-3-glucanase)	Beta 1-3-glucanase and homologous proteins/pepper, olive	Wagner, 2004 [53]
Hev b 5 (latex acid protein)	Potato, kiwi, cassava, curry	Yagami, 2009 [59]
Hev b 6.01 (pro-hevein)	Class I chitinase/banana, avocado	Radauer, 2011 [50]
Hev b 6.02 (hevein)	Class I chitinase/banana, avocado	Radauer, 2011 [50]
Hev b 7 (patatin-like)	Potato, tomato (*Solanaceae* storage protein)	Seppala, 2000 [54]
Hev b 8 (profilin- panallergen)	Profilin/pepper, celery, pineapple, grasses, birch	Takemura, 2020 [56]
Heb b 11 (class I chitinase)	Class I chitinase	Parisi, 2021 [3]
Hev b 12 (LTP-panallergen)	LTP/*Cannabis sativa*	Faber, 2015 [63]
Hev b 13 (esterase)	Potato	Parisi, 2021 [3]
Hev b 15 (serine-protease inhibitor)	PR-6	Parisi, 2021 [3]

## 5. Diagnosis of Latex Allergy 

Approximately 250 allergens have been identified in natural rubber latex, of which 15 (Hev b 1–Hev b 15) are officially included in the nomenclature list of the International Nomenclature Committee of Allergens (IUIS) [64] and recognized by the World Health Organization (WHO) [3] (see “Allergens and allergic sensitization”). Natural rubber proteins are associated with both asymptomatic sensitization and immune-mediated hypersensitivity. The prevalence of hypersensitivity associated with one or more foods in patients with latex allergy is estimated to be 60–90% [65]. The first step in diagnosing latex allergy consists in collecting an accurate clinical history that investigates the presence of other allergies, previous surgical interventions, or medical procedures involving the use of latex gloves [66]. 

As discussed above, in fact, subjects at risk are patients suffering from various pathologies including spina bifida and urogenital anomalies. Therefore, the temporal relationship between exposure to latex products and the onset of symptoms must be evaluated. The latter, as reported above in more detail (see “Clinical manifestations and cross-reactions”), are usually attributable to local reactions after contact with latex products, but symptoms/signs are also described as systemic [66]. Finally, it should be asked whether the patient has had clinical manifestations after fruit intake; in particular, banana, kiwi, figs, papaya, avocado, and chestnuts are the most frequently involved fruits in latex-related food allergy [33].

Diagnostic confirmation can be obtained in vivo with cutaneous SPT and in vitro with the dosage of specific IgE, which can be performed using various methods. The positivity of these tests indicates a sensitization to the latex. Skin prick tests are performed by applying a drop of standardized commercial extract to the skin of the flexor surface of the forearm and pricking it lightly with a special lancet [67]. 

After about 10–15 min, if a wheal ≥ 3 mm in diameter appears, the result is considered positive. In a clinical study, SPT with latex extract showed a sensitivity of about 93% and a specificity of 100% [31]. However, allergenic extracts for skin testing are not available in all countries, so the prick-by-prick test with highly allergenic latex gloves can be used as an alternative. However, this method is not standardized. Latex patch tests, on the other hand, are used to identify delayed type IV reactions. Rare cases of delayed latex allergy have been reported, and patch tests are useful for differentiating allergic contact dermatitis from irritant contact dermatitis [68]. 

In cases of suspected type IV hypersensitivity reactions such as allergic contact dermatitis, patch tests for rubber additives should be performed by placing patches on the skin between the scapulae. The plasters are removed after 48 h, and in cases of erythema and/or infiltration in the following 24–48 h, the test is considered positive. The allergens that have most commonly shown positive reactions are carbamates, thiuram mixture, 2-mercaptobenzothiazole, and 1,3-diphenylguanidine [69]. Skin tests must be performed in a hospital environment by allergists who are experts in the technique for performing the test and in the interpretation of the results, as there is a risk of inducing systemic reactions, even if overall the anaphylactic reactions reported in the literature are less than 0.02% [70]. A risk-free test, on the other hand, is the in vitro dosage of serum IgE specific for latex. This represents a specific test but is more expensive and not as readily available as skin tests. Furthermore, the serum dosage of specific IgE for latex has a sensitivity of 70–80%, whereby a positive test indicates the presence of sensitization to latex proteins; however, the percentage of cases in which it can be falsely negative is not negligible [71]. This lower diagnostic accuracy is caused by not all latex allergens being represented. The cut-off values depend on the assay method used and the population studied [72].

Component-resolved diagnostics (CRD), currently also called molecular diagnostics, helps the specialist to identify positivity for clinically relevant allergenic molecules. Sensitization to some latex components (e.g., Hev b 1, Hev b 5, Hev b 6.01, and Hev b 6.02) is associated with more severe clinical phenotypes and is expressed as genuine latex allergy, whereas sensitization to other allergens (eg Hev b 8) is generally asymptomatic or associated with milder symptoms.

Numerous specific IgE assay methods exist, among which ImmunoCAP seems to outperform ISAC microarrays, although there is good agreement between the two tests [72,73]. Other commercial tests for CRD include: Immulite, Alex MADX, Euroline, and FABER. Diagnostic tests for latex allergy are listed and explained in Table 8.

**Figure 1:**
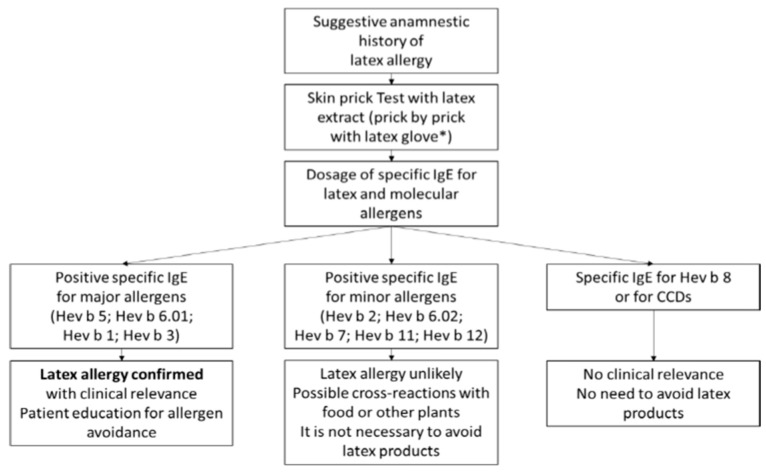
Diagnostic flow chart and prevention indications. Confirmatory diagnostic testing usually begins with skin and serum sIgE testing for native extracts. If these tests are positive, the allergenic molecules of the latex (and possibly of the CCDs) are determined, which can suggest a greater or lesser clinical relevance of the allergy (modified from [3]). * The procedure is not standardized.

Among the in vitro tests, the basophil activation test (BAT) is a functional test based on flow cytometry which evaluates the degree of basophil activation after stimulation with recombinant latex allergens. This test may theoretically be able to distinguish clinically relevant allergy from asymptomatic sensitization [74]. In order to simplify the allergy diagnostic process from a practical point of view in case of suspected latex allergy, Figure 1 (adapted from [3,75]) traces the different paths that identify subjects at risk of reaction from those with non-clinically relevant cross-sensitizations, also identifying those for which precise indications of prevention must be provided [39,75,76,77].

The provocation test remains the test of choice to confirm or exclude latex allergy. The provocation test is used when the clinical history is suggestive, but the result of SPT or specific IgE dosage is discordant [69]. Several methods of performing the challenge tests (cutaneous, muco-oral, sublingual, conjunctival, nasal, and bronchial) have been reported, although some of them have low sensitivity and many limitations are related to the procedure. The skin test, also known as the “use test”, may be performed [3]. For the sublingual, conjunctival, nasal, and bronchial tests, solutions with latex extract are used in increasing dilutions at progressively higher concentrations up to any threshold dose [3]. However, the provocation test puts the patient at risk of severe reactions such as anaphylaxis; therefore, it is to be used in selected cases in which the diagnosis is not conclusive. Furthermore, it is important to identify individuals with clinically irrelevant sensitizations in order to reduce the costs associated with unnecessary allergen avoidance measures.

## 6. Management: Prevention and Therapy 

Management of latex allergy relies on avoiding contact with natural latex products [78,79]. 

The other therapeutic strategies are represented by pharmacological therapy of acute allergic reactions, by immunotherapy, and, in selected cases, by therapy with anti-IgE biological drugs [3].

## 7. Prevention

Given the diffusion of products containing latex, the prevention of this allergy must take place on several levels, represented by primary, secondary, and tertiary prevention.

### 7.1. Primary Prevention

Primary prevention is defined as the adoption of interventions and behaviors capable of avoiding or reducing the onset and development of a disease or an adverse event upstream [80]. 

In this sense, the primary prevention of allergic sensitization to latex takes the form of reducing exposure to products containing latex [3]. It is important to underline that this prevention includes measures of interest to both the general population and individuals at increased risk of developing latex allergy.

Individual prevention lies in the identification of subjects at risk of developing latex allergy. It is reiterated that in the pediatric field the following are considered risk factors for sensitization to latex: atopy, surgical interventions during the neonatal period, and repeated dental or surgical procedures during the first years of life, in particular in the presence of anatomical anomalies/functional urogenital organs [81]. It is important to inform parents of the presence of these risk factors and, if present, implement an individual prevention plan. For example, in children with spina bifida, a population at high risk of developing latex allergy [82], the use of latex-allergen-free equipment in the operating room has been shown to reduce latex allergic sensitization in these children and, consequently, the appearance of allergic symptoms connected to it [10].

Social primary prevention refers to preventive social measures aimed at creating environments free from latex allergens, both for patients at health risk and for workers most exposed to latex. The creation of completely “latex-free” environments is currently very difficult, given the widespread use of products containing latex in homes, schools, and workplaces [83].

Latex allergy management is based on preventing contact with natural latex products [78,79]. 

Other therapeutic strategies include pharmacological therapy of acute allergic reactions, immunotherapy, and, in selected cases, therapy with biological anti-IgE drugs [3]. Among natural latex products, latex gloves are the most widely used product [84]. Therefore, the labeling of all products containing latex is fundamental in order to facilitate their immediate identification and avoidance by the allergic patient [32]. However, the legislative regulation of many states has made it possible to create “latex-safe” environments [85] thanks to the use of products with a reduced allergenic content of latex, produced through a process of deproteinization, purification, and chlorination with subsequent high-temperature washing [86,87].

An example of such legislative measures is represented by the restriction on the use of latex gloves in hospital settings, which has resulted in a reduction in the rate of latex allergy among states that have adopted this measure, as demonstrated by several studies [40,65,88,89,90,91].

Specifically, there has been a decline in the prevalence of latex sensitization in healthcare workers following the introduction of latex-free gloves (e.g., in Europe and North America). Conversely, in developing countries and in those areas where such primary prevention policies are not implemented, latex allergy continues to be a major public health problem [30,92]. 

There are valid alternatives to latex on the market, such as synthetic elastomers and Yulex gloves, made from a particular form of natural latex rubber obtained from Guayule (*Parthenium argentatum*), a native desert shrub [93,94].

In addition to the use of latex-free gloves, there are other management aspects in the hospital setting that can be implemented, if not already present (see “Tertiary prevention”).

### 7.2. Secondary Prevention

Secondary prevention is defined as the early diagnosis of a pathology, thus enabling timely intervention [80].

It is the duty of the general practitioner/pediatrician of choice (but also of other specialists involved in patient care) to refer the patient for allergy evaluation in case of suspected latex allergy. In these cases, the allergist will have to collect an accurate medical history and evaluate which diagnostic tests are necessary to formalize the diagnosis of latex allergy. In this sense attention should also be paid to the latex-fresh fruit syndrome also known as latex-associated food allergy (see “Clinical manifestations and cross-reactions”).

As far as the working environment is concerned, it is important that workers promptly report any suspected allergic reactions in order to be directed towards a timely diagnostic process. In fact, having ascertained any anamnestic connotations of suspected allergic sensitization to latex, the occupational physician must subject workers exposed to contact and inhalation of latex to serial diagnostic tests to confirm any allergic sensitization and implement the necessary prevention and/or care.

### 7.3. Tertiary Prevention

Tertiary prevention relates to the prevention and mitigation of complications of an already diagnosed disease, and often includes therapeutic measures.

The primary objective in latex allergy is the avoidance of allergic reactions and, in particular, anaphylaxis by providing the patient with an action plan for the management of allergic reactions which includes, if necessary, the prescription of an adrenaline auto-injector [3,80].

In schools, it is of fundamental importance to alert and involve school staff concerning the presence of students with a known latex allergy. Each child must be equipped with an individualized action plan and self-injectable adrenaline (if necessary) [95].

School equipment should be checked to avoid contact with materials containing latex. An identification bracelet may be useful for pediatric patients with latex allergy [96].

**Figure 2:**
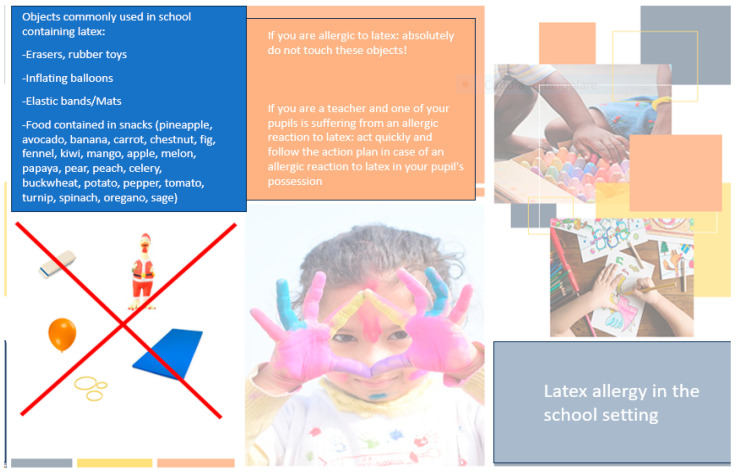
Tips for managing latex allergy in the school setting.

At a collective level, it is important to raise awareness and educate teachers and schoolmates about the creation of a latex-safe school environment through the dissemination of educational material (Figure 2) and the removal of products containing latex (e.g., balloons, erasers, materials for schoolwork, etc.) [3].

In the workplace, an individual worker education strategy must be undertaken to avoid sources of latex in the workplace [97], as well as a collective strategy aimed at the worker’s health which can lead, alternatively, to the termination of the employment relationship, the relocation of the patient in the work organization chart, or the creation of latex-safe work areas [3]. As already highlighted above, creating a latex-safe environment in a hospital environment is a difficult goal to achieve. In an observational study in a Pennsylvania hospital, 616 accidental exposures and seven allergic reactions were reported following contact with latex despite the enforcement of a latex-safe hospital regulation [3]. Consequently, it can be deduced that the creation of a completely latex-free environment, i.e., one also free from traces of latex, is very difficult in a hospital environment. At the same time, the goal of creating a latex-safe environment, through the use of products with reduced latex allergenic content and precautions aimed at avoiding patient contact with latex, is more achievable [3]. Regarding such measures, a prime example is represented by the fact that all patients suffering from latex allergy must promptly report their allergy before any medical evaluation or surgical procedure [98]. 

It is then the task of the medical and nursing staff to discuss the presence of allergies with the patient and family members before administering therapies or performing medical–surgical procedures and to possibly plan defined strategies for surgical procedures [30] and the operating list (patient allergic to latex as the first item in the list).

The adoption of strict protocols for patients affected by latex allergy and their identification by means of identification bracelets can represent a strategy to minimize the risks of inadvertent exposure to latex in such patients [3]. There are currently no unique protocols or recommendations within the different hospitals and different scientific societies [95].

Despite this heterogeneity, the shared basis of these protocols is the use of non-latex gloves, catheters, and alternative products, usually silicone, plastic, or vinyl [30].

It is desirable to set up a “Latex Allergy Task Force” in each hospital with the task of strictly regulating the presence of latex-free products in hospitals through the adoption of specific protocols [99]. This task force must include the figures most interested in the possible allergy to latex in the hospital environment (Table 9).

## 8. Immunotherapy

Immunotherapy for latex allergy was proposed and used some years ago through both the subcutaneous (SCIT) and sublingual (SLIT) routes of administration, but its efficacy has never been fully demonstrated [100], and there have been both cases of failure [101,102] and serious adverse effects [103,104]. Regarding the use of SCIT, the first case report involved a 31-year-old female with occupational exposure [105]. The first clinical trial, however, dates back to the 2000s. Despite the efficacy of desensitization, the authors reported a high incidence of systemic reactions. Subsequent studies also confirmed the high rate of adverse reactions: in a study by Tabar et al. [106], systemic reactions were even reported in 81.8% in the treatment group compared to 16.7% in the placebo group. The most studied and used immunotherapy for latex allergy is SLIT, which has demonstrated better safety and clinical and immunological efficacy results in several studies [107] (Table 10).

However, in a randomized double-blind study against placebo conducted by Gastaminza in 2011 on 28 adult patients allergic to latex, no significant differences were found in the provocation test or in vitro tests after one year of treatment with SLIT latex [102].

It should also be noted that only one [108] of the double-blind randomized controlled trials on latex SLIT was performed in the pediatric age group. In another case-control study conducted on 23 children [110], 300 ug of latex extract was sublingually administered weekly. The treated patients showed significant improvements in their latex skin prick test diameter, plasma IgG4 level, and conjunctival provocation test (after one year of SLIT), although they did not show any change in latex-specific IgE and in the test of basophil activation (BAT). A total of 28–33% of patients treated with SLIT showed mild adverse effects.

SLIT for latex can, therefore, cause adverse events; these, however, especially in children, are usually mild and of a local type [111,112]. It involves two stages. The increasing phase generally lasts four days and consists of four daily intakes, progressively increasing the extract for the first two days of treatment; then, five intakes on the third day, always progressively increasing, up to the maximum tolerated dose; and a single intake of the maximum tolerated dose on the fourth day. The maintenance phase consists of taking the maximum tolerated dose three times a week for 3–5 years. The follow-up of the patient includes an annual clinical and diagnostic evaluation (SPT or specific IgE dosage) as a control of the efficacy of the immunotherapy [104]. Other protocols have induction phases of less than 2–3 days but are associated with a greater number of side effects [104]. In specific cases, it may also be useful to tailor immunotherapy, as done in a study by Giovannini et al. [111] in which immunotherapy in SLIT mode was appropriately dosed in relation to the tolerance of a 7-year-old girl with high reactivity to latex and concomitant recurrent urticaria.

Latex SLIT is generally indicated in selected symptomatic patients in whom preventive measures are not feasible or have proved ineffective [3]. Absolute contraindications to latex SLIT include immune-based diseases, chronic heart and lung disease, renal insufficiency, treatment with beta-blockers, and hypersensitivity to one of the excipients present in the solution [109].

In general, however, it can be observed that the complexity of the clinical manifestations of latex allergy limits the power of studies performed with SLIT, because patients with different symptoms are often grouped together. Furthermore, when exposed to specific provocation tests, the cases studied are relatively few, and long-term efficacy studies after the cessation of immunotherapy are lacking [107].

Finally, it bears consideration that the last controlled study on latex SLIT [110] dates to about 10 years ago and that currently, for the best preventive measures and the consequent decrease in cases of latex allergy, this type of treatment is always less used.

## 9. Biological Drugs

Scientific evidence regarding biological drugs in the treatment of latex allergy is scarce and not definitive. The only biological drug being studied is the anti-IgE monoclonal antibody omalizumab. The only available study was published in 2004 [112].

The authors demonstrated the efficacy of omalizumab in reducing skin and ocular symptoms in workers affected by latex allergy and exposed to a working environment with the presence of latex sources. In addition to this study, there are some case reports which have highlighted the benefits of this treatment in cases of contact urticaria or latex-induced asthma [113,114].

The use of omalizumab in association with latex immunotherapy has also been proposed [30], with particular attention to the risk–benefit ratio both in the workplace with the presence of latex sources and in other environments [3].

## Figures and Tables

**Table 1:** Main commonly used objects containing latex allergens.

Main Common Use Objects Containing Latex Allergens	
Gloves	Shoes
Stickers	Diapers
Balloons	Elastic bands
Anti-slip mats	Erasers
Condoms	Pacifiers
Birth control diaphragms or caps	Sports equipment (swimming masks, googles, fins)
Bottle teats	Tires
Rubber toys	Mattresses
Stamps	Medical devices (e.g., syringes, plasters, tourniquets, eye drops, catheters, balloons, orthodontic appliances)

**Table 5:** Allergenic components of Hevea brasiliensis latex (Hev b) and their clinical relevance. Adapted from MAUG 2 (Molecular Allergology Users’ Guide 2) available online: https://hub.eaaci.org/resources_guidelines/molecular-allergology-users-guide-2-0/, accessed on 1 June 2022.

Allergen	Common Name	Molecolar Weight (kDa)	Relevance as an Allergen for Latex Glove Users	Relevance as an Allergen for Spina Bifida
Hev b 1	Rubber elongation factor β-1,3-glucanase	14	Minor	Major
Hev b 2	β-1,3-glucanase	34	Minor	Minor
Hev b 3	Small rubber protein particle	24	Minor	Major
Hev b 4	Lecithinase homologue	53–55	Minor	Minor
Hev b 5	Acidic structural protein	16	Major	Major/Minor
Hev b 6.01	Prohevein (precursor of hevein Hev b 6.02, the major IgE binding domain)	20	Major	Minor
Hev b 7	Patatin-like protein (esterase) from latex-B- and C-serum (two isoforms: Hev b 7.01 and Hev b 7.02)	44	Minor	Minor
Hev b 8	Profilin (actin-binding protein) (several isoforms and variants)	14	Minor	Minor
Hev b 9	Enolase	51	Minor	Minor
Hev b 10	Manganese superoxide dismutase	26	Minor	Minor
Hev b 11	Class I chitinase	30	Minor	Minor
Hev b 12	Non-specific lipid transfer protein type 1 (nsLTP1)	9	Minor	Minor
Hev b 13	Esterase	42	Minor	Minor
Hev b 14	Hevamine	30	Minor	Minor
Hev b 15	Serine protease inhibitor	7.5	Minor	Minor

**Table 6:** Involved mechanisms of adverse reactions to latex (modified from [3]).

Mechanism Type	Type of Reaction	Clinical Manifestations
IgE-mediated	Type I—immediate	Urticaria, angioedema, rhinitis, conjunctivitis, bronchial obstruction, anaphylaxis, etc.
Non-IgE-mediated	Type IV—delayed	Hyperemia and dry skin, erythema, pruritus, vesicular lesions (may extend beyond the contact site), etc.
Non-immunological	Irritative reaction	Skin hyperemia, pruritus, dryness (does not extend beyond the contact site), etc.

**Table 8:** Diagnostic tests for latex allergy (adapted from [3]).

Type of Test	Test	Description
Skin tests	Skin prick tests	Method of choice to confirm or exclude latex allergy, with commercial extracts or PbP technique (puncture of the skin through the glove)
	Patch tests	Delayed hypersensitivity reactions are most often attributed to additives
Laboratory test	Specific IgE for the extract	ImmunoCAP and IMMULITE (sensitivity >80%; specificity >95%)
	Component resolved diagnostics (CRD) ImmunoCAP ISAC microarray (Thermo Fisher Scientific, Phadia, Uppsala, Sweden) Immulite (Siemens Healthcare Diagnostics Inc., Erlangen, Germany) Alex MADX (MacroArray Diagnostics GmbH Vienna, Austria) Euroline (EUROIMMUN AG, Lübeck, Germany) FABER (Allergy Data Laboratories S.r.l Latina, Italy)	rHev b 1, rHev b 3, rHev b 5, rHev b 6, rHev b 8, CCD
	BAT	Basophil activation after stimulation with recombinant latex allergens. It may help to distinguish clinically relevant sensitization from asymptomatic sensitization.
Provocation testing (in case of suggestive history but negative skin and laboratory tests)	“Use test” with latex glove	Latex glove first on a finger for 15 min–2 h. In the absence of symptoms, the entire glove can be slipped onto the hand. The negative control is performed by placing a nitrile or vinyl glove on the other hand. The test is considered positive when itching, erythema, vesicles, or respiratory symptoms appear.
	Rub test	It can provide false positive results and it is not standardized. Very low diagnostic power, it is not used.
	Specific bronchial provocation test	Aqueous latex extract that is sprayed or roomed with aerosolized glove extracts or shaken latex gloves to create aerosols of the powder. Pulmonary function tests and the appearance of bronchial symptoms are then evaluated.
	Nasal and conjunctival provocation test	Used in some studies, but it has little meaning.

**Table 9:** Healthcare professionals to be involved in the “Latex Allergy Task Force” in each healthcare company.

Public Administration	Medical Directors	Medical Doctors	Nurses	Pharmacists
Executive Director	Chief of the Internal Medicine Department	Attendings of the Internal Medicine Department	Chief of Nurses of the Internal Medicine Department	Chief of Pharmacy
Health Director	Chief of the Emergency Department, Chief of the Allergy and Immunology Department	Attendings of the Emergency Department, Attendings of the Allergy and Immunology Department	Chief of Nurses of the Emergency Department	
Marketing Chief	Chief of the Surgery Department	Attendings of the Surgery Department	Chief of Nurses of the Surgery Department	
Hospital Manager	Chief of the Anesthesiology and Perioperative Care Department	Attendings of the Anesthesiology and Perioperative Care Department	Chief of Nurses of the Anesthesiology and Perioperative Care Department	

**Table 10:** Double-blind randomized controlled trials of latex SLIT (modified from [107]).

Bibliography	Methods and Main Results
Bernardini et al., 2006 [108]	A total of 20 pediatric patients. Administration of SLIT latex (210 ug weekly). Treated patients showed significant improvements in the use of latex gloves and rubbing-tests, even if they did not show any changes in latex-specific IgE and in latex skin prick tests. No adverse effects were reported during SLIT.
Nettis et al., 2007 [109]	A total of 35 patients. Administration of SLIT latex (300 ug weekly). Treated patients showed significant improvements in the use of latex gloves, the bronchial provocation test, the symptoms questionnaire, needed rescue therapy, and the diameter of the latex SPT (after one year of SLIT), even if they did not show any changes in latex-specific IgE and in latex skin prick tests. A total of 17% of patients treated with SLIT showed mild adverse effects.
Gastaminza et al., 2011 [102]	A total of 28 patients. Administration of SLIT latex (210 ug weekly). Treated patients did not show significant improvements in the measured outcomes. Four treated patients showed mild adverse effects.

## Data Availability

Not applicable.
